# IQGAP1 Is a Phosphotyrosine-Regulated Scaffold for SH2-Containing Proteins

**DOI:** 10.3390/cells12030483

**Published:** 2023-02-02

**Authors:** Louise Thines, Zhigang Li, David B. Sacks

**Affiliations:** Department of Laboratory Medicine, National Institutes of Health, Bethesda, MD 20892, USA

**Keywords:** Abl, IQGAP1, MET, phosphorylation, phosphotyrosine, receptor tyrosine kinase, scaffold protein, SH2, signaling

## Abstract

The scaffold protein IQGAP1 associates with over 150 interactors to influence multiple biological processes. The molecular mechanisms that underly spatial and temporal regulation of these interactions, which are crucial for proper cell functions, remain poorly understood. The receptor tyrosine kinase MET phosphorylates IQGAP1 on Tyr^1510^. Separately, Src homology 2 (SH2) domains mediate protein–protein interactions by binding specific phosphotyrosine residues. Here, we investigate whether MET-catalyzed phosphorylation of Tyr^1510^ of IQGAP1 regulates the docking of SH2-containing proteins. Using a peptide array, we identified SH2 domains from several proteins, including the non-receptor tyrosine kinases Abl1 and Abl2, that bind to the Tyr^1510^ of IQGAP1 in a phosphorylation-dependent manner. Using pure proteins, we validated that full-length Abl1 and Abl2 bind directly to phosphorylated Tyr^1510^ of IQGAP1. In cells, MET inhibition decreases endogenous IQGAP1 phosphorylation and interaction with endogenous Abl1 and Abl2, indicating that binding is regulated by MET-catalyzed phosphorylation of IQGAP1. Functionally, IQGAP1 modulates basal and HGF-stimulated Abl signaling. Moreover, IQGAP1 binds directly to MET, inhibiting its activation and signaling. Collectively, our study demonstrates that IQGAP1 is a phosphotyrosine-regulated scaffold for SH2-containing proteins, thereby uncovering a previously unidentified mechanism by which IQGAP1 coordinates intracellular signaling.

## 1. Introduction

IQGAP1 is a scaffold protein with a multidomain architecture that allows for interactions with numerous diverse proteins [1]. By facilitating the physical assembly of protein complexes, IQGAP1 regulates multiple cellular activities, ranging from cytoskeletal dynamics [2,3] to intracellular signaling [4,5]. Tight spatial and temporal control of IQGAP1 scaffolding properties in response to various stimuli is required to elicit adequate cellular responses. However, the molecular mechanisms that modulate the binding of IQGAP1 to interactors remain mostly unexplored.

MET is the receptor tyrosine kinase for hepatocyte growth factor (HGF) [6]. MET activation by HGF initiates signaling cascades, such as the mitogen-activated protein kinase (MAPK) and phosphatidylinositol-3 kinase (PI3K)/Akt pathways, to modulate cell growth, proliferation, survival, and motility [6]. While MET activity is required for normal physiological processes, including embryogenesis and wound healing, aberrant HGF/MET signaling drives tumorigenesis [7,8]. Interestingly, a few studies have established connections between HGF/MET signaling and IQGAP1. For example, HGF stimulates the association of IQGAP1 with some of its binding partners. HGF increases the binding of IQGAP1 to the Rac/Cdc42 guanine nucleotide exchange factor Asef, the actin-binding protein cortactin, and the microtubule-binding protein EB1 to enhance endothelial barrier function [9,10]. Moreover, HGF enhances the binding of IQGAP1 to the Ser/Thr protein kinase PAK6 to promote disassembly of cell–cell adhesions [11], suggesting that IQGAP1 links HGF signaling to cellular processes. Importantly, we documented that activated MET catalyzes phosphorylation of IQGAP1 on Tyr^1510^ [12]. Mutation of Tyr^1510^ of IQGAP1 into non-phosphorylatable Ala affects the ability of HGF to promote Akt activation and cell migration, suggesting that phosphorylation of the Tyr^1510^ of IQGAP1 by MET alters cell function [12].

Protein phosphorylation can initiate protein–protein interactions, where a protein specifically binds to the newly phosphorylated residue of another protein to mediate functional outcomes [13]. A classic example of this is the recruitment of signaling proteins that contain intrinsic Src homology 2 (SH2) domains, which dock onto phosphorylated tyrosine residues in specific target sequences [14]. The recruitment of SH2-containing proteins is a key mechanism in signal transduction by receptor and non-receptor tyrosine kinases [14,15]. Among the 110 human SH2-containing proteins are the non-receptor tyrosine kinases Abl1 and Abl2. Both Abl1 and Abl2 regulate actin dynamics, but each also has separate functions. Abl1 mediates DNA damage repair via a unique DNA-binding domain, whereas Abl2 has additional actin-binding capacity that enhances its cytoskeletal remodeling functions [16,17]. 

In this study, we show that IQGAP1 binds directly to MET receptor tyrosine kinase, and that this interaction impairs MET signaling. Moreover, we demonstrate that MET-catalyzed phosphorylation of IQGAP1 creates a docking site for several SH2 domains, including those of Abl1 and Abl2. These associations have functional implications, as IQGAP1 modulates Abl kinase activity. More generally, this work identifies for the first time that IQGAP1 is a phosphotyrosine-regulated scaffold for SH2-containing proteins, which uncovers an additional mechanism by which it contributes to intracellular signaling.

## 2. Materials and Methods

### 2.1. Antibodies and Reagents

Roswell Park Memorial Institute (RPMI) 1640, fetal bovine serum (FBS), Alexa Fluor^TM^ 488 phalloidin, lipofectamine RNAiMAX, and Halt protease and phosphatase inhibitors were purchased from Thermo Fisher Scientific (Waltham, MA, USA). Purified active MET (the intracellular region of MET, comprising amino acids 974–1390) was obtained from Millipore Sigma (St. Louis, MO, USA). Protein A-Sepharose and glutathione-Sepharose were obtained from GE Healthcare (Chicago, IL, USA). Crizotinib was purchased from Selleck Chemicals (Houston, TX, USA). IQGAP1 siRNA (sc-35700), control siRNA (sc-37007), and normal rabbit and mouse IgG were purchased from Santa Cruz Biotechnology (Dallas, TX, USA). Recombinant human Abl1 and Abl2 were obtained from Abcam (Cambridge, UK). Recombinant human HGF was obtained from R&D Systems (Minneapolis, MN, USA). The primary antibodies used in this study are in Table S1. Secondary antibodies were purchased from LI-COR (Lincoln, NE, USA).

### 2.2. Cell Culture and Treatments

H1993 and HepG2 cells were purchased from the American Type Culture Collection (Manassas, VA, USA). Cells were cultured in RPMI 1640 containing 10% FBS at 37 °C in 5% CO_2_. For HGF treatments, cells were serum-starved for 16 h and then incubated with 50 ng/mL HGF for the time indicated in figure legends in serum-free medium. Where indicated, the cell-permeable MET inhibitor crizotinib was added to the cells 24 h before cell lysis at a concentration of 100 nM. Transfections were carried out with IQGAP1 siRNA or control siRNA using lipofectamine RNAiMAX as instructed by the manufacturer.

### 2.3. Construction and Expression of Plasmids

The plasmids coding for the GST-tagged SH2 domains of Abl1 and Abl2 were kindly provided by the Protein Array and Analysis Core (MD Anderson Center, Houston, TX, USA). These constructs and GST-IQGAP1 were expressed in *Escherichia coli* essentially as previously described [18]. Briefly, induction of expression was carried out with 100 μM iso-propyl-β-D-thiogalactoside at 37 °C. After 3 h, bacteria were lysed by sonication in PBS (pH 7.2) containing 2 mM EDTA, 1 mM phenylmethylsulfonyl fluoride, and 10 mM dithiothreitol (DTT). After removal of cell debris (15,000× *g* for 10 min at 4 °C), lysates were applied onto glutathione-Sepharose columns. The columns were washed with PBS containing 10 mM DTT before recovery of the beads. The purity and molecular masses of the GST fusion proteins were examined by heating the samples bound to the beads for 5 min at 95 °C in Laemmli sample buffer for subsequent SDS-PAGE and Coomassie blue staining.

### 2.4. In Vitro Phosphorylation of IQGAP1

IQGAP1 was phosphorylated in vitro by pure active MET kinase essentially as previously described [12]. Briefly, GST-IQGAP1 purified on glutathione-Sepharose was incubated in kinase buffer (50 mM HEPES, pH 7.3, 5 mM MgCl_2_, 10 mM DTT, 1 mM EDTA, 1% glycerol). Subsequently, 0.2 mM ATP and 1 μg active recombinant human MET were added to initiate the reaction. Control reactions were carried out under the same conditions by omitting ATP. After incubating for 30 min, the reaction was quenched by immediately putting the samples on ice and washing the beads with PBS (2 min at 4 °C). Phosphorylation of IQGAP1 was verified by Western blotting using anti-phosphorylated Tyr and anti-IQGAP1 antibodies. 

### 2.5. Pull-Down Analyses

Binding analyses of pure MET, Abl1, and Abl2 were performed by incubating 2 μg of recombinant human proteins with 1 μg of GST-IQGAP1 or GST alone, both on glutathione-Sepharose beads, at 4 °C for 3 h. The buffer contained 50 mM Tris-HCl (pH 7.4), 150 mM NaCl, and 1% Triton X-100 (Buffer A) and was supplemented with protease and phosphatase inhibitors. After the beads were washed five times with Buffer A, bound proteins were resolved by SDS-PAGE followed by Western blotting. Blots were probed with anti-MET, anti-Abl1, anti-Abl2, and anti-IQGAP1 primary antibodies, followed by fluorescent-labeled secondary antibodies. Imaging was performed with the Odyssey CLx infrared imaging system (LI-COR, Lincoln, NE, USA).

For binding of endogenous IQGAP1 to the SH2 domains of Abl1 or Abl2, H1993 cells were lysed in Buffer A containing protease and phosphatase inhibitors. Centrifugation for 10 min at 15,000× *g* and 4 °C was used to pellet insoluble fractions. After pre-clearing with glutathione-Sepharose beads for 1 h at 4 °C, equal amounts of protein lysates were incubated with the SH2 domains of Abl1/2 purified on glutathione-Sepharose for 3 h at 4 °C. Proteins bound to the beads were analyzed by SDS-PAGE and Western blotting.

### 2.6. Immunoprecipitations

Cells were washed with PBS at 4 °C and lysed as described in the preceding paragraph. For IQGAP1 precipitation, pre-cleared lysates were incubated at 4 °C for 3 h with anti-IQGAP1 polyclonal antibodies previously bound onto protein A beads. For all other immunoprecipitations, monoclonal anti-MET, anti-Abl1, or anti-Abl2 antibodies were added to the cell lysates for 3 h at 4 °C, followed by a 2 h incubation with protein A-Sepharose beads. Controls for immunoprecipitations with polyclonal and monoclonal antibodies were non-immune rabbit serum and IgG, respectively. After washing the beads five times with Buffer A, bound proteins were resolved by SDS-PAGE and processed by Western blotting. 

### 2.7. Proximity Ligation Assay

Proximity ligation assays (PLAs) were performed essentially as previously described [19]. Briefly, after seeding cells on coverslips in 24-well plates, they were fixed with 4% paraformaldehyde. Next, 0.25% Triton X-100 was added to permeabilize the cells. After blocking for 2 h with 10% FBS, cells were incubated for 16 h with anti-MET and anti-IQGAP1 antibodies at 4 °C. Oligonucleotide-labeled secondary antibodies, namely donkey anti-mouse PLUS and anti-rabbit MINUS PLA probes (Millipore Sigma, St. Louis, MO, USA), were added for 1 h at 37 °C. The Duolink^®^ in situ detection reagent Red (Millipore Sigma) was used for DNA ligation and amplification. Actin staining was performed with Alexa Fluor^TM^ 488 phalloidin. Incubation with individual primary antibodies served as negative controls. Slide mounting medium was used to mount coverslips. Cells were analyzed with a Zeiss LSM 880 confocal microscope (Carl Zeiss, Oberkochen, Germany) with a 63x objective lens (numerical aperture 14). The number of PLA dots per cell was quantified using Fiji/ImageJ (NIH, Bethesda, MD, USA). Individual cells were segmented based on actin staining. The PLA dots, segmented using the red fluorescence intensity above background, were then counted for each cell.

### 2.8. SH2 Domain Microarray

Analysis was performed at the Protein Array and Analysis Core (MD Anderson Center, Houston, TX, USA). The peptide array used for the analysis contained the GST-tagged SH2 domains from 124 different proteins, each spotted in duplicate into eight grids. GST alone is present in the middle of each grid as a negative control. To generate the array, the SH2 domains were cloned into the pGEX-4T1 vector and synthesized by the Biomatik Corporation (Kitchener, Canada). Expression and purification of recombinant proteins were carried out essentially as previously described [20]. The GST-tagged constructs, and GST alone, were then spotted onto nitrocellulose-coated glass slides using the Aushon 2470 Microarrayer (Quanterix) (Grace Bio-Labs, Bend, OR, USA). Equal loading of the constructs was verified by probing the array with an anti-GST primary antibody and fluorescent dye-conjugated secondary antibodies.

Biotinylated IQGAP1 peptides (amino acids 1502 to 1518) with either non-phosphorylated or phosphorylated Tyr^1510^ were synthesized by Thermo Fisher Scientific. The peptides were conjugated to streptavidin-Cy3 fluorophore for use as microarray probes. After incubation for 1 h at 22 °C in blocking buffer (PBS-Tween containing 3% milk and 3% BSA), the arrays were incubated with fluorophore-conjugated peptides for 16 h at 4 °C in blocking buffer. The microarrays were washed with PBS-Tween, dried, and fluorescence from the bound peptides was detected using the InnoScan 1100 AL Fluorescence Scanner (Innopsys, Chicago, IL, USA) [20]. 

### 2.9. Statistical Analyses

GraphPad Prism 9 was used for all statistical analyses. The statistics used to analyze each data set are described in the figure legends. Where indicated, Image Studio 2.0 (LI-COR, Lincoln, NE, USA) was used to quantify pertinent bands from Western blots.

## 3. Results 

### 3.1. IQGAP1 Binds Directly to the MET Receptor

**Figure 1:**
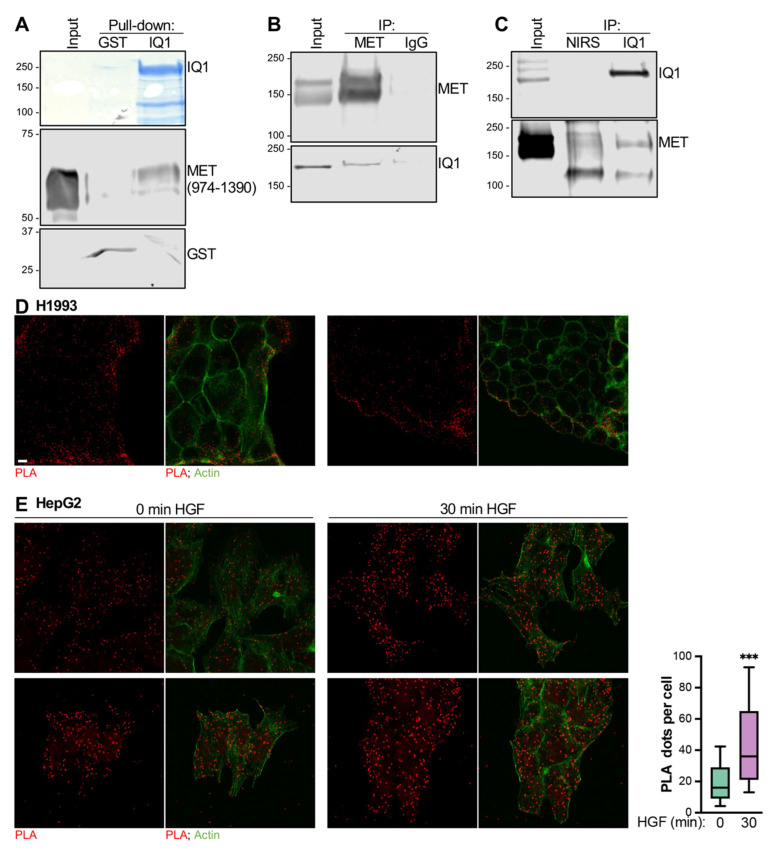
IQGAP1 binds directly to the MET receptor. (**A**). GST-IQGAP1 (IQ1), bound to glutathione-Sepharose beads, was incubated with 2 μg of the purified intracellular region of MET (residues 974–1390). Control pull-downs were carried out with GST-glutathione-Sepharose (GST). After washing, proteins attached to the beads were eluted in Laemmli sample buffer and analyzed by SDS-PAGE. The gel was cut at ∼ 80 kDa. The top portion of the gel was stained with Coomassie blue. The lower portion was processed by Western blotting and probed with anti-MET and anti-GST antibodies. Input designates pure MET not subjected to pull-down. The positions of migration of molecular weight markers are indicated on the left. All images are from the same gel. The data shown are representative of two independent experiments. The multiple bands observed on the MET blot likely reflect partial degradation. (**B**). H1993 cells were cultured and lysed. Equal amounts of protein from cell lysates were subjected to immunoprecipitation (IP) with an anti-MET antibody. Control precipitation was carried out with mouse IgG. Samples were resolved by SDS-PAGE followed by Western blotting and probed with anti-MET and anti-IQGAP1 (IQ1) antibodies. Unfractionated cell lysate (Input) was processed in parallel. Both images are from the same membrane. (**C**). Immunoprecipitation was carried out from H1993 cell lysates as described for (**B**), except anti-IQGAP1 antibody was used. Control precipitation was carried out with non-immune rabbit serum (NIRS). Western blots were probed with anti-IQGAP1 (IQ1) and anti-MET antibodies. Both images are from the same membrane. All blots in panels (**B**,**C**) are representative of three independent experiments. Data presented in panels (**A–C**) were cropped from the full immunoblots shown in Figure S1B. (**D**). H1993 cells grown on coverslips were fixed, permeabilized, and incubated with anti-MET and anti-IQGAP1 antibodies. A proximity ligation assay (PLA) was carried out using Duolink in situ probes and detection reagents (Millipore Sigma). Cell images were acquired via confocal microscopy. Red spots indicate positive PLA signals. Actin was stained with phalloidin (green). Scale bar, 10 μm. Two representative images from two independent experiments (80 cells each) are shown. (**E**). HepG2 cells were incubated in the absence (left panels) or presence (right panels) of HGF. A PLA was performed as described for (**D**), and foci were quantified from confocal images using ImageJ. The boxplot shows the distribution of the number of PLA dots per cell observed with or without HGF (horizontal line, median; box, 25–75% percentiles; whiskers, 10–90% percentiles; n = 100). Statistical analysis was carried out using an unpaired *t*-test (***, *p* ≤ 0.001).

The MET receptor catalyzes phosphorylation of IQGAP1 on Tyr^1510^ [12]. However, the possible association of IQGAP1 with MET had not been studied. Here, we investigated whether IQGAP1 binds MET. We incubated GST-IQGAP1, purified on glutathione-Sepharose, with purified MET (the intracellular region of MET, comprising amino acids 974–1390). We verified by Western blotting that MET is autophosphorylated on Tyr^1234/1235^, indicating that it is the activated form of MET (Figure S1A). Western blotting revealed that activated intracellular MET binds directly to GST-IQGAP1 (Figure 1A). The absence of MET from samples incubated with GST alone validates that binding is specific (Figure 1A). These data show that IQGAP1 binds directly to the intracellular portion of active MET.

Immunoprecipitation was used to determine if the endogenous proteins interact in cells. Analysis was performed with non-small cell lung cancer H1993 cells, which constitutively overexpress active MET [21]. We immunoprecipitated endogenous MET from H1993 cell lysates. Western blotting reveals that IQGAP1 co-immunoprecipitates with MET (Figure 1B). To confirm binding, we performed the reverse immunoprecipitation. IQGAP1 was immunoprecipitated from H1993 cell lysates, and blots were probed for MET. MET co-immunoprecipitates with IQGAP1 (Figure 1C). In both cases, the absence of signals from the negative control precipitations (IgG and NIRS for MET and IQGAP1 immunoprecipitations, respectively) verifies the specificity of the interaction. These results demonstrate that endogenous IQGAP1 and MET form a complex in H1993 cell lysates. 

We further analyzed the interaction between endogenous IQGAP1 and endogenous MET in H1993 intact cells using a proximity ligation assay (PLA). In this assay, cells are incubated with primary antibodies specific to IQGAP1 and MET. Close proximity (≤40 nm) between the antibodies is required for generation of a signal (red dots). Consistent with our immunoprecipitation results, a positive PLA signal was observed, showing that IQGAP1 and MET are in close vicinity in H1993 intact cells (Figure 1D). 

To ascertain whether HGF, the cognate ligand of MET, modulates MET:IQGAP1 interaction, we carried out similar PLA experiments in HepG2 cells incubated with or without HGF. As in H1993 cells, we observed positive PLA signals (Figure 1E), reflecting that the close MET:IQGAP1 proximity is not restricted to cells that overexpress MET. Importantly, incubating cells with HGF for 30 min significantly increases (by 2.2-fold) the number of PLA dots, implying that HGF stimulates the formation of MET:IQGAP1 complexes in HepG2 cells (Figure 1E). Minimal signals were observed for cells incubated with each primary antibody separately (Figure S2), validating that the data in Figure 1D,E identify close localization between MET and IQGAP1.

### 3.2. IQGAP1 Impairs HGF-Stimulated MET Activation and Signaling

**Figure 2:**
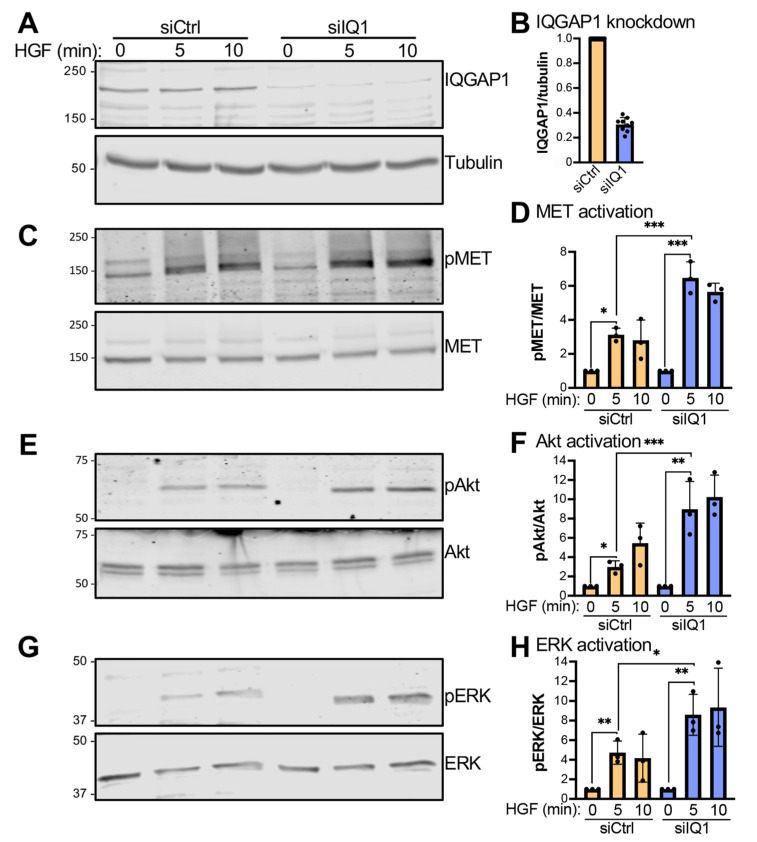
Knockdown of IQGAP1 enhances MET activation and signaling. HepG2 cells were transfected with control siRNA (siCtrl) or IQGAP1 siRNA (siIQ1). Next, 48 h after transfection, cells were starved of serum for 16 h and then incubated with 50 ng/mL HGF for 0, 5, or 10 min. Equal amounts of cell lysates were analyzed by SDS-PAGE and Western blotting. (**A**). The membrane was probed with an anti-IQGAP1 antibody. An anti-tubulin antibody was used for the loading control. (**B**). The intensity of the IQGAP1 band was quantified using Image Studio 2.0 (LI-COR Biosciences) and corrected for that of tubulin in the corresponding sample. Data are expressed as the IQGAP1/tubulin ratio from the three HGF timepoints for three independent repetitions (mean ± SD, n = 9). The ratio observed for siCtrl cells was set as 1. (**C**). Antibodies specific to phosphorylated MET (pMET, Tyr^1234^/Tyr^1235^) and total MET were used to probe the Western blot membrane. (**D**). The pMET bands were quantified and corrected for the amount of total MET in the corresponding sample. (**E**). The membrane was probed with antibodies to phosphorylated Akt (pAkt, Ser^473^) and total Akt. (**F**). The pAkt and Akt signals were quantified and the pAkt/Akt ratio was calculated. (**G**). Phosphorylated ERK (pERK, Thr^202^/Tyr^204^) and total ERK were detected on the Western blot membrane using specific antibodies. (**H**). The pERK bands were quantified and corrected for the amount of total ERK in the corresponding sample. Graphs D, F, and H show the means ± SD from three independent experiments. For these graphs, the ratio of phosphorylated to total protein in the absence of HGF (0 min) was set as 1 for both siCtrl and siIQ1 cells. Statistical analyses were performed using unpaired *t*-tests (*, *p* ≤ 0.05; **, *p* ≤ 0.01; ***, *p* ≤ 0.001). All blots in this figure are representative of three independent experiments. The full blots of all three replicates are shown in Figure S3.

Binding of IQGAP1 to growth factor receptors often modulates their signaling [22,23,24]. Therefore, we investigated whether the interaction of IQGAP1 with MET influences HGF-stimulated MET activation and signaling. We knocked down IQGAP1 in HepG2 cells using siRNA. Control transfections with scrambled siRNA were carried out in parallel. Western blotting shows that IQGAP1 siRNA reduces the amount of endogenous IQGAP1 protein by 69 ± 5% (mean ± SD) (Figure 2A,B).

HGF activates MET by inducing its autophosphorylation on Tyr^1234^/Tyr^1235^ [25]. We evaluated the phosphorylation of these two residues in HepG2 cells incubated with HGF for 0, 5, or 10 min. HGF significantly augments the phosphorylation of MET on Tyr^1234^/Tyr^1235^ in both control and IQGAP1 siRNA-treated cells (Figure 2C,D), reflecting activation. Interestingly, HGF-stimulated activation of MET was up to 2.1-fold higher in IQGAP1 knockdown cells than in control cells (Figure 2C,D). These data suggest that IQGAP1 prevents full activation of MET by HGF.

We also examined signaling pathways activated by MET, namely the PI3K/Akt and MAPK cascades. Analysis was performed by evaluating Akt and ERK phosphorylation, respectively. We observed that HGF significantly increases activation of Akt in both control and IQGAP1 knockdown HepG2 cells (Figure 2E,F). Analogous to MET activation, the extent of HGF-stimulated Akt phosphorylation was 2.4-fold greater in cells in which IQGAP1 was knocked down than that measured in control cells (Figure 2E,F). HGF also stimulates ERK activation in control and IQGAP1 knockdown HepG2 cells (Figure 2G,H). Consistent with the effects on MET and Akt, knockdown of IQGAP1 resulted in 2.1-fold greater activation of ERK by HGF (Figure 2G,H). Together, our results imply that IQGAP1 impairs the ability of HGF to fully activate MET and its signaling to both the PI3K/Akt and MAPK cascades.

### 3.3. Phosphorylation of Tyr^1510^ of IQGAP1 Creates a Docking Site for SH2 Domains

**Figure 3:**
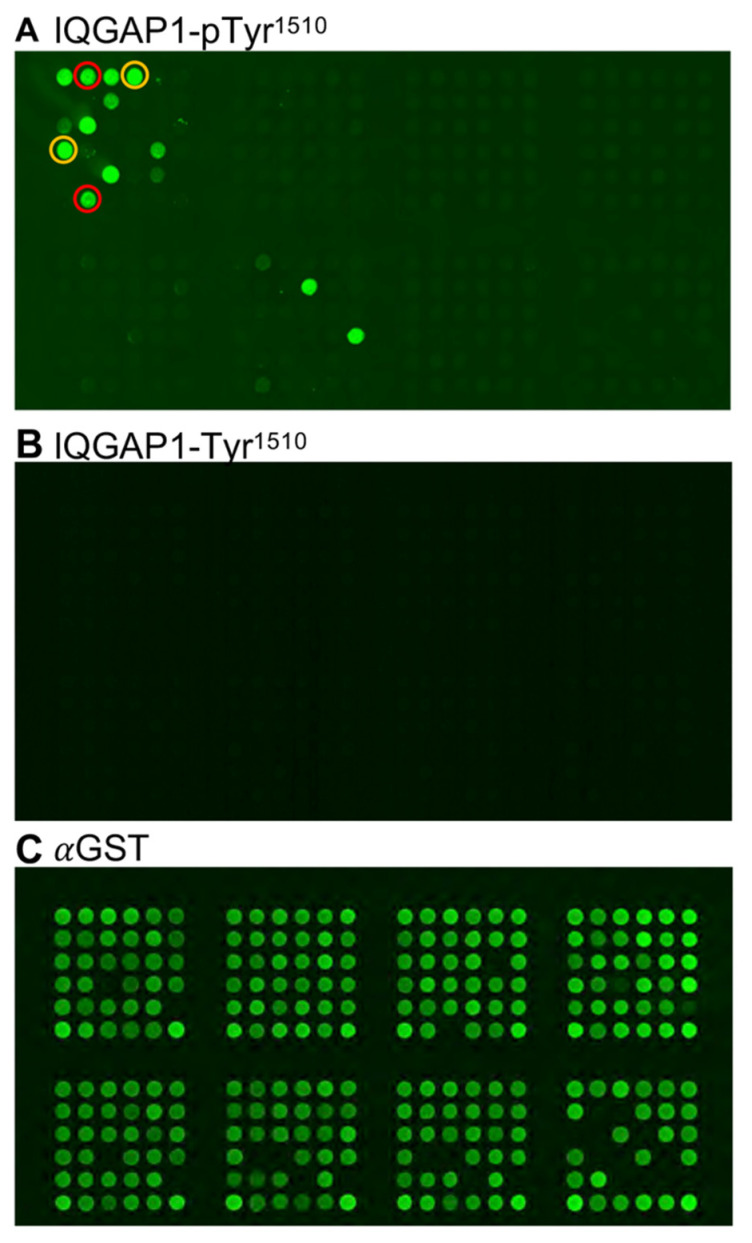
Phosphorylation of Tyr^1510^ of IQGAP1 creates a docking site for SH2 domains. (**A**). A peptide array containing 124 different GST-tagged recombinant SH2 protein domains, each spotted in duplicate, was generated as detailed in *Materials and Methods*. GST alone was spotted on the array as the negative control. Biotinylated peptides comprising residues 1502–1518 of IQGAP1 with phosphorylated Tyr^1510^ (pTyr^1510^) were labeled with fluorescent streptavidin and incubated with the array for 16 h at 4 °C. After washing, fluorescence from the bound peptides (green dots) was detected. The red and orange circles delineate the duplicates for the SH2 domains of Abl1 and Abl2, respectively. (**B**). Similar analysis was conducted with the same IQGAP1 peptide, except Tyr^1510^ was not phosphorylated. (**C**). The peptide array was probed with anti-GST antibody to show the positions and loading of the GST-SH2 domains and control GST.

The functional consequences of MET-catalyzed phosphorylation of the Tyr^1510^ of IQGAP1 remain poorly characterized. It is well known that SH2 domains specifically dock onto phosphorylated tyrosine (pTyr) residues of their binding partners to mediate signaling outcomes [15]. Thus, we set out to determine whether specific SH2 domains would bind to pTyr^1510^ of IQGAP1. To do so, we used a peptide array coated with the GST-tagged SH2 domains of 124 different proteins. We incubated the array with a synthetic peptide comprising amino acids 1502 to 1518 of IQGAP1 with pTyr^1510^. This peptide bound to the SH2 domain of eight proteins (Figure 3A, green dots). Minimal binding to GST alone confirms specificity. To evaluate whether the docking of these eight SH2 domains on IQGAP1 is dependent on phosphorylation of Tyr^1510^, we incubated the SH2 array with the same IQGAP1 peptide, except with non-phosphorylated Tyr^1510^. None of the SH2 domains bound to the non-phosphorylated peptide (Figure 3B). Anti-GST antibody was used to show equal loading of the arrayed SH2 domains (Figure 3C). These data indicate that phosphorylation of Tyr^1510^ of IQGAP1 creates a docking site for selected SH2 domains. 

### 3.4. The SH2 Domains of Abl1 and Abl2 Dock on pTyr^1510^ of Endogenous IQGAP1

**Figure 4:**
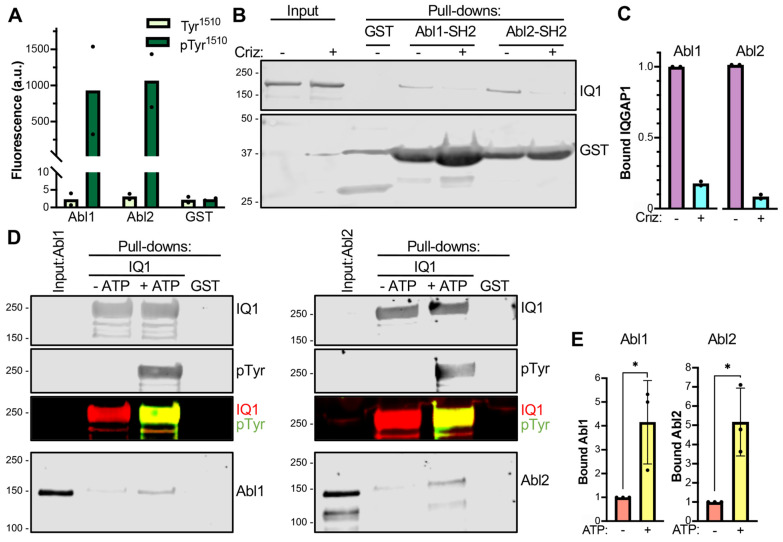
The SH2 domains of Abl1 and Abl2 bind directly to tyrosine-phosphorylated IQGAP1. (**A**). Quantification of the fluorescence intensity of the IQGAP1 peptides with unphosphorylated (Tyr^1510^, pale green bars) or phosphorylated (pTyr^1510^, dark green bars) Tyr^1510^ bound to the SH2 domains of Abl1 or Abl2 on the SH2 array. Binding to GST is the control. Data represent the mean fluorescence from two duplicates. a.u., arbitrary units. (**B**). H1993 cells were incubated with 100 nM crizotinib (criz, *+*) or vehicle DMSO (−). After 24 h, cells were lysed and equal amounts of protein lysate were incubated with the purified GST-SH2 domains of Abl1 or Abl2 bound to glutathione-Sepharose. Control pull-downs were carried out with GST-glutathione-Sepharose. After washing, proteins attached to the beads were eluted in Laemmli sample buffer and analyzed by Western blotting. The membrane was probed with anti-IQGAP1 (IQ1) and anti-GST antibodies. *Input* designates unfractionated cell lysates. Both panels are from the same membrane. Blots are representative of two independent experiments. The full blots of the two replicates are shown in Figure S6. (**C**). The IQGAP1 bands observed after pull-down were quantified using Image Studio 2.0 (LI-COR Biosciences). The intensity of IQGAP1 in DMSO-treated cells was set as 1. Data are presented as the means of two independent replicates. (**D**). Purified GST-IQGAP1 (IQ1) on glutathione-Sepharose was incubated with purified active MET in the presence (+ATP) or absence (−ATP) of ATP. After washing, beads were incubated with 2 μg of purified Abl1 (left panel) or Abl2 (right panel). Control pull-downs were carried out with GST-Sepharose. After washing, proteins attached to the beads were eluted in Laemmli sample buffer and analyzed by SDS-PAGE and Western blotting. The membrane was probed with anti-IQGAP1 (IQ1), anti-phosphotyrosine (pTyr), and anti-Abl1 or anti-Abl2 antibodies. The overlap between IQGAP1 (red) and pTyr (green) signals is visible in the merged image (yellow). Input designates pure Abl1 or Abl2 not subjected to pull-down. The blots are representative of three independent experiments. The full blots of the three replicates are shown in Figure S7. (**E**). The Abl1 and Abl2 bands observed after pull-down by GST-IQGAP1 were quantified using Image Studio 2.0 (LI-COR Biosciences). The intensity of Abl1 and Abl2 signals observed with non-phosphorylated IQGAP1 (−ATP) was set as 1. Data are the means ± SD of three independent experiments. Statistical analyses were performed with unpaired *t*-tests (*, *p* ≤ 0.05).

The SH2 domains of the non-receptor tyrosine kinases Abl1 and Abl2 were among those identified using the SH2 array. The intensities of binding between the IQGAP1 peptides and the arrayed SH2 domains of Abl1 and Abl2 are depicted in Figure 4A. The extent of binding of the non-phosphorylated peptide is less than 0.5% of that of the tyrosine-phosphorylated peptide. Peptide binding to GST was minimal (Figure 4A). These data indicate that the SH2 domains of Abl1 and Abl2 dock on Tyr^1510^ of IQGAP1 in a phosphorylation-dependent manner. 

We next investigated the binding of the SH2 domain of Abl1 to pTyr^1510^ of full length endogenous IQGAP1. To do so, we expressed a GST fusion construct of the SH2 domain of Abl1 (GST-Abl1-SH2) in *E. coli* and purified it on glutathione-Sepharose. This yields a large quantity of pure SH2 peptide (Figure S4). H1993 cells were selected for binding studies because IQGAP1 is constitutively phosphorylated on Tyr^1510^ in this cell line [12] (Figure S5). We incubated purified GST-Abl1-SH2 with H1993 cell lysates. Western blotting revealed that endogenous IQGAP1 is pulled down by GST-Abl1-SH2 (Figure 4B). Similar analysis was performed with the GST-tagged SH2 domain of Abl2 (GST-Abl2-SH2) purified on glutathione-Sepharose (Figure S4). Analogous to the SH2 domain of Abl1, GST-Abl2-SH2 binds to endogenous IQGAP1 in H1993 cell lysates (Figure 4B). The absence of IQGAP1 binding to GST alone validates specificity (Figure 4B).

To evaluate whether binding of Abl1 is influenced by MET-catalyzed phosphorylation of IQGAP1 on Tyr^1510^, we used the cell-permeable MET inhibitor crizotinib. We initially verified that crizotinib markedly attenuates tyrosine phosphorylation of IQGAP1 in H1993 cells (Figure S5), as we previously validated by mass spectrometry [12]. GST-Abl1-SH2 pulls down 82% less endogenous IQGAP1 upon crizotinib treatment (Figure 4B,C). Similarly, crizotinib decreases by 92% the amount of endogenous IQGAP1 that binds GST-Abl2-SH2 (Figure 4B,C). These data reveal that the SH2 domains of Abl1 and Abl2 bind endogenous IQGAP1, and that binding is regulated by phosphorylation of Tyr^1510^ of IQGAP1. 

### 3.5. Full-Length Abl1 and Abl2 Bind Directly to IQGAP1 Phosphorylated by MET

The aforementioned binding studies were restricted to the SH2 domains of the Abl kinases. Next, we investigated whether full-length Abl1 and Abl2 bind to phosphorylated Tyr^1510^ of IQGAP1. To do so, we used GST-IQGAP1 purified on glutathione-Sepharose. We first phosphorylated GST-IQGAP1 on the beads using pure active MET in the presence of ATP. We performed a duplicate assay where ATP was omitted to obtain non-phosphorylated IQGAP1 as a control. We validated that MET induces tyrosine phosphorylation of IQGAP1 in the presence, but not in the absence, of ATP through Western blotting (Figure 4D).

We incubated tyrosine-phosphorylated and non-phosphorylated GST-IQGAP1 with purified Abl1. GST-IQGAP1 phosphorylated in vitro by MET binds Abl1. Binding of Abl1 to non-phosphorylated GST-IQGAP1 was markedly less (Figure 4D,E). We performed similar pull-downs with purified Abl2. As with Abl1, Abl2 binds to phosphorylated IQGAP1, with little binding to the non-phosphorylated protein (Figure 4D,E). Neither Abl1 nor Abl2 bound to GST alone, validating specificity (Figure 4D). These results demonstrate that phosphorylation of IQGAP1 by MET on Tyr^1510^ governs direct binding to full-length Abl1 and Abl2, thereby validating the data of our peptide array screen.

### 3.6. Endogenous Abl1 and Abl2 Bind to Endogenous IQGAP1 with Phosphorylated Tyr^1510^

**Figure 5:**
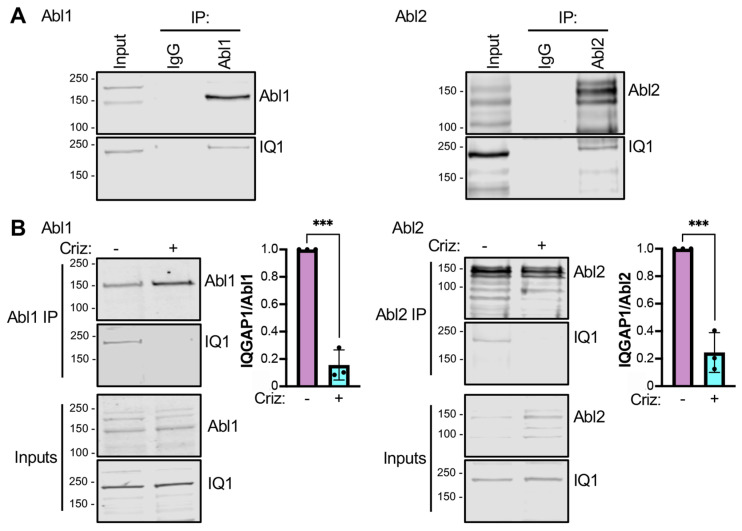
Endogenous IQGAP1 with phosphorylated Tyr^1510^ binds to endogenous Abl1 and Abl2. (**A**). H1993 cells were cultured and lysed. Equal amounts of protein from cell lysates were subjected to immunoprecipitation (IP) with anti-Abl1 (left panel) or anti-Abl2 (right panel) antibodies. Control precipitations were carried out with mouse IgG. Samples were resolved by SDS-PAGE followed by Western blotting and probed with antibodies to IQGAP1 (IQ1) and Abl1 or Abl2. Unfractionated cell lysates (Input) were processed in parallel. Both images in each panel are from the same membrane. Blots are representative of three independent experiments. The presented data were cropped from the full immunoblots shown in Figure S8. (**B**). H1993 cells were incubated with 100 nM crizotinib (criz, +) or vehicle DMSO (−) for 24 h. Cells were then lysed, Abl1 or Abl2 was immunoprecipitated, and samples were resolved by Western blotting as described for panel A. The amount of IQGAP1 was quantified and corrected for the amount of immunoprecipitated Abl1 or Abl2 in the same sample. Ratios were set as 1 for DMSO-treated (−) cells. Data are the means ± SD of three independent repetitions. Statistical analyses were performed with unpaired *t*-tests (***, *p* ≤ 0.001). All blots shown in this figure are representative of three independent experiments. The full blots of the three replicates are shown in Figure S9.

Next, we examined whether endogenous IQGAP1 binds endogenous Abl1 and Abl2 in cells. Analysis was performed by immunoprecipitation. H1993 cells were lysed and Abl1 or Abl2 was immunoprecipitated. Western blotting shows that endogenous IQGAP1 co-immunoprecipitates with endogenous Abl1 and Abl2 (Figure 5A). No signal was detected in samples precipitated with IgG, thereby confirming that the interactions are specific (Figure 5A). These data indicate that endogenous Abl1 and Abl2 bind to endogenous IQGAP1 in H1993 cells.

We investigated whether binding of Abl1 and Abl2 to endogenous IQGAP1 is regulated by phosphorylation of Tyr^1510^. We compared the amounts of endogenous IQGAP1 that co-immunoprecipitate with endogenous Abl1 and Abl2 from H1993 cells incubated with the MET inhibitor crizotinib or the vehicle. Crizotinib decreases by 84% and 75% the amount of IQGAP1 that co-immunoprecipitates with Abl1 and Abl2, respectively (Figure 5B). Consistent with our in vitro binding analyses, these data indicate that the interaction of endogenous Abl1 and Abl2 with endogenous IQGAP1 is modulated by phosphorylation of IQGAP1 on Tyr^1510^.

### 3.7. IQGAP1 Regulates Abl Functions

**Figure 6:**
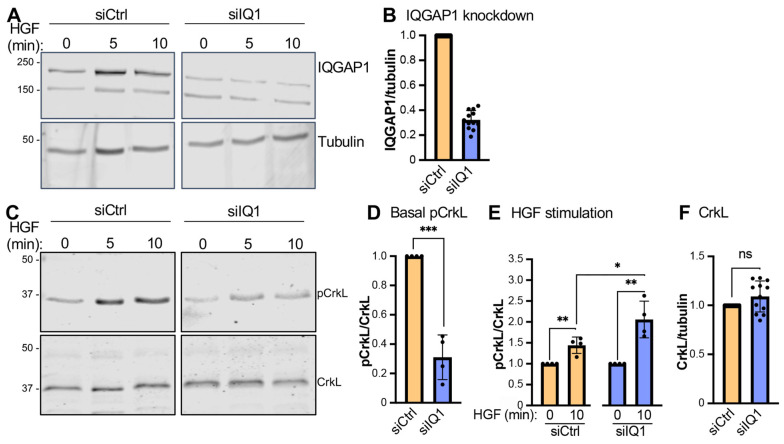
IQGAP1 modulates Abl kinase activity. HepG2 cells were transfected with scrambled siRNA (siCtrl) or siIQGAP1 (siIQ1) RNA. After 48 h, cells were starved for 16 h before being incubated with 50 ng/mL HGF for 0, 5, or 10 min. (**A**). Cells were lysed and equal amounts of protein lysates were analyzed by Western blotting using anti-IQGAP1 and anti-tubulin antibodies. (**B**). IQGAP1 signal intensity was quantified with Image Studio 2.0 (LI-COR Biosciences) and corrected for the amount of tubulin in the corresponding sample. Data are expressed as the IQGAP1/tubulin ratio from the three HGF timepoints for four independent repetitions (mean ± SD, n = 12). The ratio in siCtrl cells was set as 1. (**C**). Western blots of the same HepG2 cell lysates as those used for panel A were probed for CrkL phosphorylated on Tyr^207^ (pCrkL) and total CrkL. (**D**). The pCrkL band at the 0 min HGF timepoint (basal phosphorylation) was quantified and corrected for the amount of total CrkL in the corresponding sample. siCtrl cells were set at 1 (mean ± SD, n = 4). (**E**). The pCrkL/CrkL ratio was calculated for the 0 and 10 min HGF timepoints. The ratio for 0 min HGF was set at 1 for both siCtrl and siIQ1 cells to visualize the effect of HGF stimulation on pCrkL (mean ± SD, n = 4). (**F**). The CrkL band was quantified and corrected for the amount of tubulin in the same sample. Data are expressed as the IQGAP1/tubulin ratio from the three HGF timepoints for four independent repetitions (mean ± SD, n = 12). All blots shown in this figure are representative of four independent experiments. Statistical analyses were carried out using unpaired *t*-tests (*, *p* ≤ 0.05; **, *p* ≤ 0.01; ***, *p* ≤ 0.001). The full blots of the four replicates are shown in Figure S10.

The pTyr-dependent binding of Abl1 and Abl2 to IQGAP1 prompted us to investigate whether IQGAP1 influences Abl signaling. Abl1 and Abl2 catalyze phosphorylation of the adaptor protein CrkL on Tyr^207^ [26]. Therefore, we measured phosphorylation of CrkL as a readout of Abl kinase activity. Analysis was performed in HepG2 cells with IQGAP1 knockdown. Consistent with the data in Figure 2A, knockdown by siRNA reduces IQGAP1 by 71 ± 6% (mean ± SD) (Figure 6A,B).

Since HGF activates the Abl kinases [27], we incubated the control and IQGAP1 knockdown cells with HGF to stimulate Abl-catalyzed phosphorylation of CrkL. We observed that IQGAP1 knockdown decreases by 69% the basal phosphorylation of CrkL, which was observed in the absence of HGF (Figure 6C,D), suggesting that IQGAP1 facilitates Abl signaling to CrkL in non-stimulated cells. HGF significantly increases the phosphorylation of CrkL in both control and IQGAP1 knockdown cells (Figure 6C,E), reflecting activation of the Abl kinases. Importantly, the HGF-stimulated increase in CrkL phosphorylation is 1.4-fold greater in IQGAP1 knockdown cells than in control cells (Figure 6C,E), indicating that IQGAP1 impairs HGF-stimulated Abl signaling. IQGAP1 knockdown does not significantly modify the abundance of total CrkL (Figure 6C,F), verifying that the effects of IQGAP1 knockdown on pCrkL are due to modulation of phosphorylation.

## 4. Discussion

**Figure 7:**
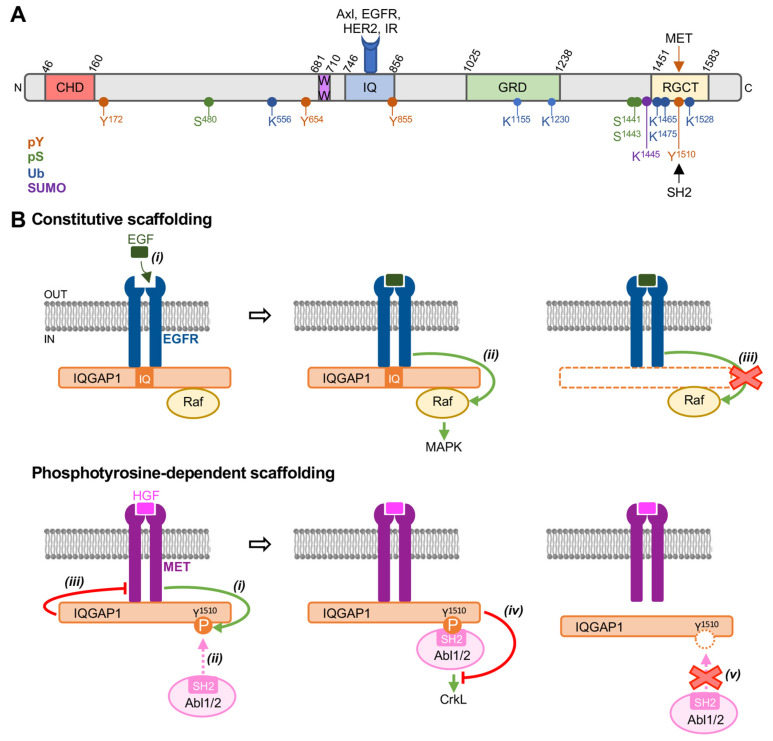
Model of IQGAP1 in receptor tyrosine kinase signaling. (**A**). Schematic of IQGAP1 that highlights known post-translational modifications. The five domains are CHD (calponin-homology domain), WW, IQ, GRD (GAP-related domain), and RGCT (RasGAP_C-terminus). Post-translational modifications that have been characterized on IQGAP1 are shown below the modified amino acid. The identified domain to which receptor tyrosine kinases bind is depicted above IQGAP1. Phosphorylation of IQGAP1 on Tyr^1510^, leading to the recruitment of SH2 domains, is also shown. (**B**). Model depicting the identified modes of action of IQGAP1 in receptor tyrosine kinase signaling. Upper panel: constitutive scaffolding by IQGAP1. IQGAP1 binds constitutively to both the growth factor receptor and a downstream signaling protein. (i) Ligand binding activates the receptor. (ii) Scaffolding by IQGAP1 facilitates activation of the signaling protein by the activated receptor, initiating downstream signaling. (iii) Signal transduction from the activated receptor to the effector protein does not occur in the absence of scaffolding by IQGAP1. The mode of action depicted here is for the EGF receptor (EGFR) and B-Raf kinase in activation of the MAPK cascade [23,46]. Lower panel: phosphotyrosine-dependent scaffolding by IQGAP1. (i) Phosphorylation of tyrosine on IQGAP1 by an activated receptor tyrosine kinase (ii) initiates recruitment of selected SH2-containing proteins. The mechanism illustrated here is for the MET receptor tyrosine kinase and the SH2-containing proteins Abl1 and Abl2. In this example, IQGAP1 (iii) impairs MET activation and signaling and (iv) decreases HGF-stimulated signaling of Abl to the adaptor protein CrkL. Therefore, IQGAP1 functions as a rheostat regulating the flux of signaling between activated MET receptors and SH2-containing signaling proteins. (v) Recruitment of SH2-containing proteins does not occur in the absence of receptor tyrosine kinase-catalyzed phosphorylation of IQGAP1. Abbreviations: EGF, epidermal growth factor; EGFR, EGF receptor; HER2, human epidermal growth factor receptor 2; HGF, hepatocyte growth factor; IR, insulin receptor; P, phosphate; pS, serine phosphorylation; pY, tyrosine phosphorylation; SUMO, SUMOylation; Ub, ubiquitination.

Post-translational modifications influence various protein characteristics, including activity, stability, localization, and conformation [28]. Numerous post-translational modifications, ranging from serine [29,30] and tyrosine phosphorylation [12] to ubiquitination [31] and SUMOylation [32], have been identified on IQGAP1 (Figure 7A). Interestingly, some of these post-translational modifications regulate IQGAP1 scaffolding properties. For example, ubiquitination of IQGAP1 on Lys^1155^ and Lys^1230^ alters its binding to the Rho GTPases Cdc42 and Rac1, with consequences on Cdc42 activation and cell migration [31]. Similarly, analysis with peptides containing phosphomimetic mutations suggests that phosphorylation of the Ser^1441^ and Ser^1443^ of IQGAP1 impairs the binding of Cdc42 [33,34]. Tyrosine phosphorylation is a post-translational modification that commonly initiates protein–protein interactions by recruiting interactors that contain pTyr recognition domains [13]. However, none of the previously identified tyrosine phosphorylation events on IQGAP1 had been shown to regulate its binding properties. 

This gap in the literature prompted us to evaluate whether tyrosine phosphorylation of IQGAP1 modulates the binding of selected proteins. We focused on Tyr^1510^ because it is the only tyrosine residue phosphorylated on IQGAP1 for which the responsible kinase has been identified [12]. We confined our investigation to proteins that contain SH2 domains, the largest class of domains that recognize pTyr [15]. Our SH2 peptide array screen demonstrates that Tyr^1510^ of IQGAP1 binds the isolated SH2 domains of several proteins in a phosphorylation-dependent manner. These data reveal for the first time that tyrosine phosphorylation of IQGAP1 initiates the recruitment of SH2 domains. 

The SH2 domains of the non-receptor tyrosine kinases Abl1 and Abl2 were among those that docked to Tyr^1510^ of IQGAP1 in a phosphorylation-dependent manner in our peptide array screen. Since the array assessed only isolated SH2 domains, we evaluated whether full-length Abl proteins bind to IQGAP1. We showed that pure IQGAP1 phosphorylated in vitro by MET kinase binds pure full-length Abl1 and Abl2; binding to non-phosphorylated IQGAP1 was minimal. Moreover, the interaction of endogenous Abl1 and Abl2 with endogenous IQGAP1 was detected in H1993 cells, which constitutively overexpress active MET [21]. Importantly, decreasing phosphorylation of IQGAP1-Tyr^1510^ by crizotinib markedly impairs IQGAP1:Abl associations in this cell line. Collectively, these data indicate that phosphorylation of Tyr^1510^ on IQGAP1 catalyzed by the MET receptor tyrosine kinase promotes SH2-mediated binding of the Abl kinases to IQGAP1. These findings augment the results of our SH2 peptide array. It is noteworthy that the binding to pTyr^1510^, which is part of a Y-A-A-L motif, is consistent with the observation that the Abl kinases dock specifically to phosphotyrosine residues in Y-x-x-[P/L/V] sequences [35]. Published studies have identified the binding of IQGAP1 to several SH2-containing proteins, including PI3K [36], SOCS3 [37], ShcA [38], Src [39], and STAT1/3 [40]; however, none of these interactions was shown to be modulated by tyrosine phosphorylation. Therefore, our work identifies Abl kinases as the first proteins whose interaction with IQGAP1 is regulated by tyrosine phosphorylation. 

To investigate whether IQGAP1 regulates Abl1 and Abl2 functions, we evaluated the phosphorylation of one of their substrates, namely the adaptor protein CrkL. IQGAP1 knockdown impairs basal phosphorylation of CrkL, implying that IQGAP1 promotes basal CrkL phosphorylation catalyzed by Abl. This observation could potentially result from the stimulation of Abl intrinsic kinase activity by IQGAP1. Although CrkL binding to IQGAP1 has not been documented, another possibility is that IQGAP1 scaffolds both the Abl kinases and CrkL to facilitate phosphorylation. In contrast to unstimulated cells, IQGAP1 knockdown increases HGF-induced CrkL phosphorylation, suggesting that IQGAP1 impairs HGF-stimulated Abl signaling. This result is likely to be a consequence of the reduction produced by IQGAP1 in MET activation stimulated by HGF that we observed in this study, which in turn reduces HGF-stimulated Abl activation. Together, our data demonstrate that IQGAP1 modulates both basal and growth factor-stimulated Abl signaling. The potential consequences of IQGAP1 on the biological functions of Abl proteins, namely cytoskeletal dynamics and DNA damage repair [41], remain to be addressed. Interestingly, we previously documented that replacement of Tyr^1510^ of IQGAP1 with non-phosphorylatable Ala enhances HGF-stimulated cell migration [12]. In light of the roles of Abl1 and Abl2 in cell migration [41], it is tempting to speculate that the effects on cell migration of this IQGAP1 mutation may, at least in part, be caused by abrogation of Abl:IQGAP1 interaction.

Phosphorylation of Tyr^1510^ of IQGAP1 is catalyzed by the MET receptor [12]. While connections between MET and IQGAP1 have been documented [9,10,11,12], no previous study showed binding of the two proteins. Here, we reveal that IQGAP1 and MET form a complex in cells. In HepG2 cells, HGF increases the PLA signal between MET and IQGAP1, implying that IQGAP1 binds HGF-activated MET to a greater extent than inactivated MET. In vitro analyses with pure proteins further demonstrate that purified IQGAP1 binds directly to the purified intracellular portion of activated MET.

Since the interaction of IQGAP1 with growth factor receptors often influences ligand-induced signaling [1], we examined whether IQGAP1 alters HGF/MET signaling. IQGAP1 knockdown in HepG2 hepatocellular carcinoma cells significantly increases HGF-stimulated MET activation and signaling to Akt and ERK. Consistent with our results, IQGAP1 knockdown in Snu-449 hepatocellular carcinoma cells was recently reported to enhance HGF-stimulated MET activation and phosphorylation of Akt [42]. Taken together, these findings demonstrate that IQGAP1 attenuates full activation of HGF/MET signaling. Several potential mechanisms could explain how IQGAP1 impairs HGF-stimulated MET activation. IQGAP1 binding could modify the conformation of the kinase domain of MET, thereby altering ATP binding. Alternatively, IQGAP1 could reduce the affinity of MET toward HGF by inducing conformational changes in the extracellular portion of MET. IQGAP1 could also prevent MET homodimerization, which is required for its trans-autophosphorylation and activation. We previously made analogous observations with the Axl receptor; IQGAP1 inhibits Gas6-stimulated activation of Axl and Gas6/Axl signaling to Akt [22]. Nevertheless, IQGAP1 more commonly augments signaling by the cell surface receptors with which it associates. For example, direct binding of IQGAP1 to the epidermal growth factor receptor (EGFR) [23], human epidermal growth factor receptor-2 [24], insulin receptor [43], and platelet-derived growth factor receptor-β [44] enhances ligand-stimulated signal transduction. Moreover, IQGAP1 scaffolds kinases in the PI3K/Akt [36] and MAPK [45] pathways, which facilitates maximal activation of Akt and ERK by insulin and EGF, respectively. Combined, these results suggest that the scaffolding functions of IQGAP1 in receptor signaling, particularly in the MAPK and PI3K/Akt pathways, differ in response to cell stimulation by different ligands.

To date, the roles of IQGAP1 in growth factor signaling have been generally documented to result from the constitutive scaffolding of receptors and signaling molecules by IQGAP1, independent of tyrosine phosphorylation. A well-characterized example is the role of IQGAP1 in EGFR-mediated activation of Raf kinase (Figure 7B). IQGAP1 binds to both EGFR [23] and Raf [46] in cells. Activation of EGFR upon the binding of EGF stimulates Raf activity, which in turn initiates activation of the MAPK cascade. EGF is unable to activate Raf in the absence of IQGAP1 [46], demonstrating that scaffolding by IQGAP1 is required for EGF-stimulated signaling to Raf (Figure 7B). Importantly, we failed to identify tyrosine phosphorylation of IQGAP1 through mass spectrometry upon cell treatment with EGF [23], confirming that this mechanism is independent of tyrosine phosphorylation of IQGAP1. The current study suggests an alternative phosphotyrosine-regulated mechanism by which IQGAP1 influences growth factor signaling. We reveal here that tyrosine phosphorylation of IQGAP1 by activated MET receptor tyrosine kinase initiates recruitment of SH2-containing proteins, including the non-receptor tyrosine kinases Abl1 and Abl2, to determine signaling outputs. IQGAP1 inhibits HGF-stimulated MET and Abl signaling, suggesting that it functions as a rheostat regulating the flux of signaling between growth factor stimulation and SH2-containing proteins. Importantly, recruitment of the Abl proteins does not occur in the absence of phosphorylation of IQGAP1 by MET receptor tyrosine kinase (Figure 7B). An analogous mode of action has been documented for other scaffold/adaptor proteins. For example, tyrosine phosphorylation of Shc by EGFR enables SH2-mediated recruitment of Grb2, which in turn initiates activation of the Ras-MAPK pathway [47]. Similarly, the insulin receptor catalyzes tyrosine phosphorylation of insulin receptor substrate adaptor proteins, which recruit PI3K and Grb2 via their SH2 domains to stimulate the PI3K/Akt and MAPK pathways, respectively [48]. These studies, combined with our data presented here, emphasize the fundamental role of the phosphotyrosine-dependent recruitment of SH2 proteins by scaffolds in receptor signaling.

In conclusion, this work demonstrates the binding of IQGAP1 to MET, with functional consequences for both proteins. Moreover, it identifies for the first time that IQGAP1 is a phosphotyrosine-regulated scaffold for SH2-containing proteins. Altogether, our study provides molecular insight into the regulation of signaling pathways and expands the mechanisms by which IQGAP1 scaffolds proteins to modulate intracellular signaling cascades.

## Data Availability

The data are contained within the article and supplementary material.

## Supplementary Materials

The following supporting information can be downloaded at: https://www.mdpi.com/article/10.3390/cells12030483/s1, Figure S1: Supporting data to Figure 1; Figure S2: Negative controls for the IQGAP1:MET proximity ligation assay; Figure S3: Raw immunoblots of Figure 2; Figure S4: Purification of the GST-tagged SH2 domains of Abl1 and Abl2; Figure S5: Crizotinib decreases MET-catalyzed tyrosine phosphorylation of IQGAP1 in H1993 cells; Figure S6: Raw immunoblots of Figure 4B; Figure S7: Raw immunoblots of Figure 4D; Figure S8: Raw immunoblots of Figure 5A; Figure S9: Raw immunoblots of Figure 5B; Figure S10: Raw immunoblots of Figure 6; Table S1: Antibodies used in this study.
