# Supplementary material for: IQGAP1 Is a Phosphotyrosine-Regulated Scaffold for SH2-Containing Proteins
Source: Cells. 2023 Feb 2;12(3):483. doi: 10.3390/cells12030483 (PMC9913818; doi:10.3390/cells12030483)
Supplement: Supplementary file 1 [file cells-12-00483-s001.zip › cells-1960454-supplementary.pdf]

Supplementary Materials

Table S1. Antibodies used in this study.

Protein detected	Source / Reference	Dilution
Abl1	Cell Signaling Technology, 2862S	1:1000 (WB)
	Santa Cruz Biotechnology, sc-23	1:100 (IP)
Abl2	Abcam, ab134134	1:1000 (WB)
	Santa Cruz Biotechnology, sc-81154	1:100 (IP)
Akt	Cell Signaling Technology, 2920S	1:1000 (WB)
CrkL	Santa Cruz Biotechnology, sc-365092	1:1000 (WB)
ERK	Cell Signaling Technology, 9107S	1:1000 (WB)
GST	Santa Cruz Biotechnology, sc-138	1:1000 (WB)
IQGAP1	Millipore, 05-504	1:100 (PLA)<br>1:1000 (WB)
	Antiserum [18]	1:100 (IP)<br>1:1000 (WB)
MET	Cell Signaling Technology, 8198S	1:100 (PLA)<br>1:1000 (WB)
	Santa Cruz Biotechnology, sc-514148	1:100 (IP)
pAkt (Ser^{473})	Cell Signaling Technology, 4060S	1:1000 (WB)
pCrkL (Tyr^{207})	Cell Signaling Technology, 3181S	1:1000 (WB)
pERK (Thr^{202}/Tyr^{204})	Cell Signaling Technology, 4377S	1:1000 (WB)
pMET (Tyr^{1234}/Tyr^{1235})	Cell Signaling Technology, 3077S	1:1000 (WB)
pTyr	Cell Signaling Technology, 8954S	1:2000 (WB)
Tubulin	Sigma, T5201	1:1000 (WB)

WB: Western blotting, IP: immunoprecipitation, PLA: proximity ligation assay.

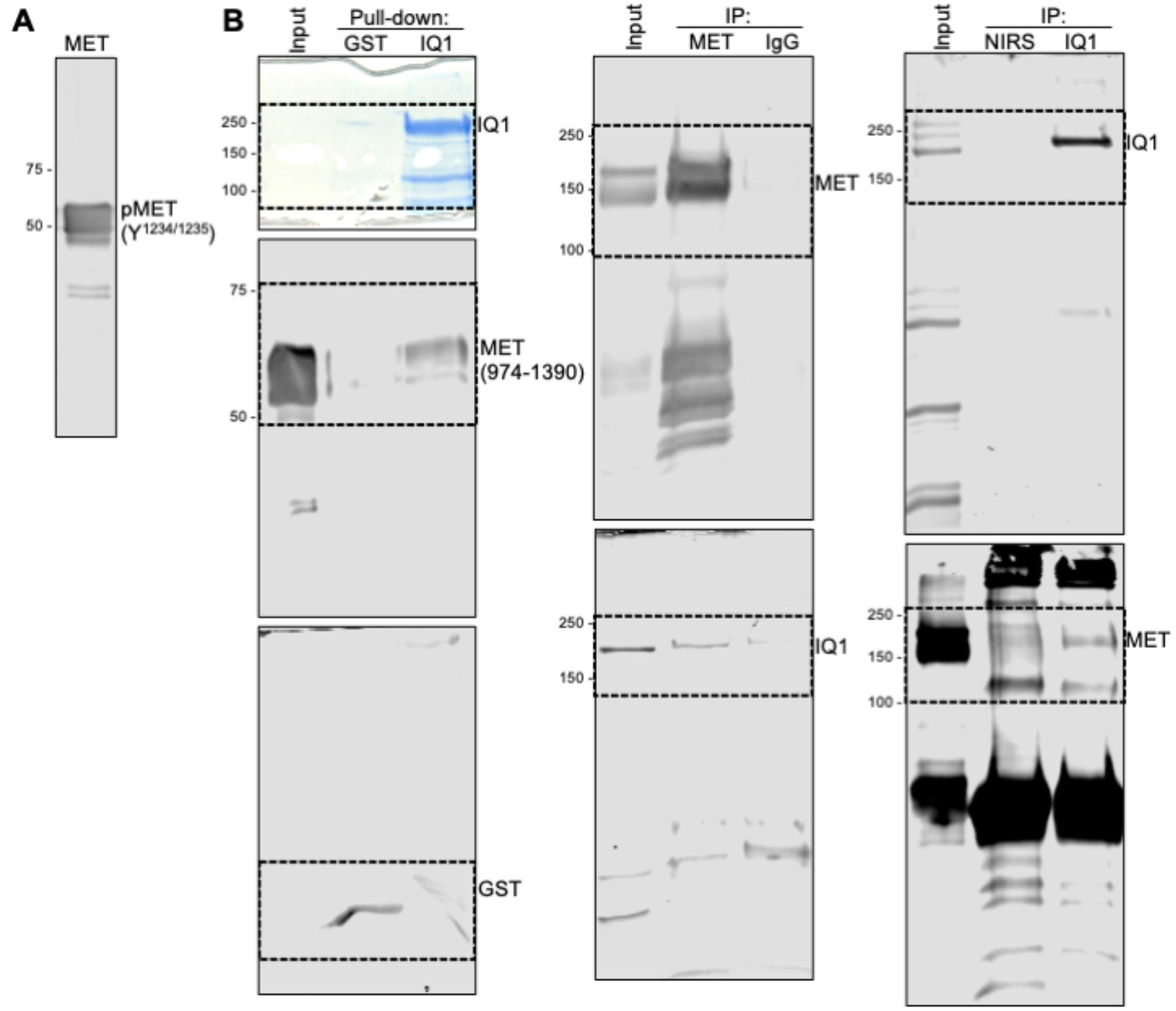

Figure S1. Supporting data to Figure 1. A. The purified intracellular portion of MET (Sigma) was analyzed by Western blotting with anti-phosphoMET antibodies recognizing its autophosphorylation sites pTyr$^{1234/1235}$. **B.** Raw Coomassie blue-stained gel and immunoblots of Figure 1. The dotted rectangles delineate the areas shown in Figure 1.

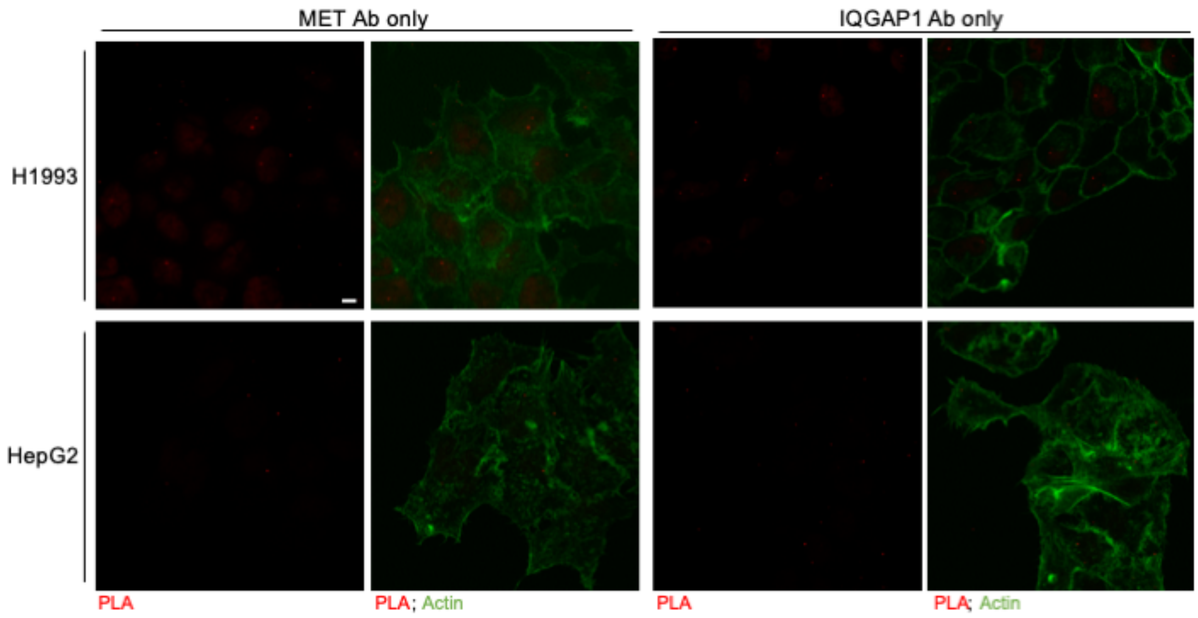

Figure S2. Negative controls for the IQGAP1:MET proximity ligation assay. H1993 (top panel) or HepG2 (bottom panel) cells grown on coverslips were fixed, permeabilized, and incubated separately with either anti-MET (MET Ab only) or anti-IQGAP1 (IQGAP1 Ab only) antibodies. Proximity ligation assay (PLA) was carried out using the Duolink *in situ* probes and detection reagents (Sigma-Aldrich). Cell images were acquired by confocal microscopy. Red spots indicate positive PLA. Actin was stained with phalloidin (green). Scale bar, 10 μm.

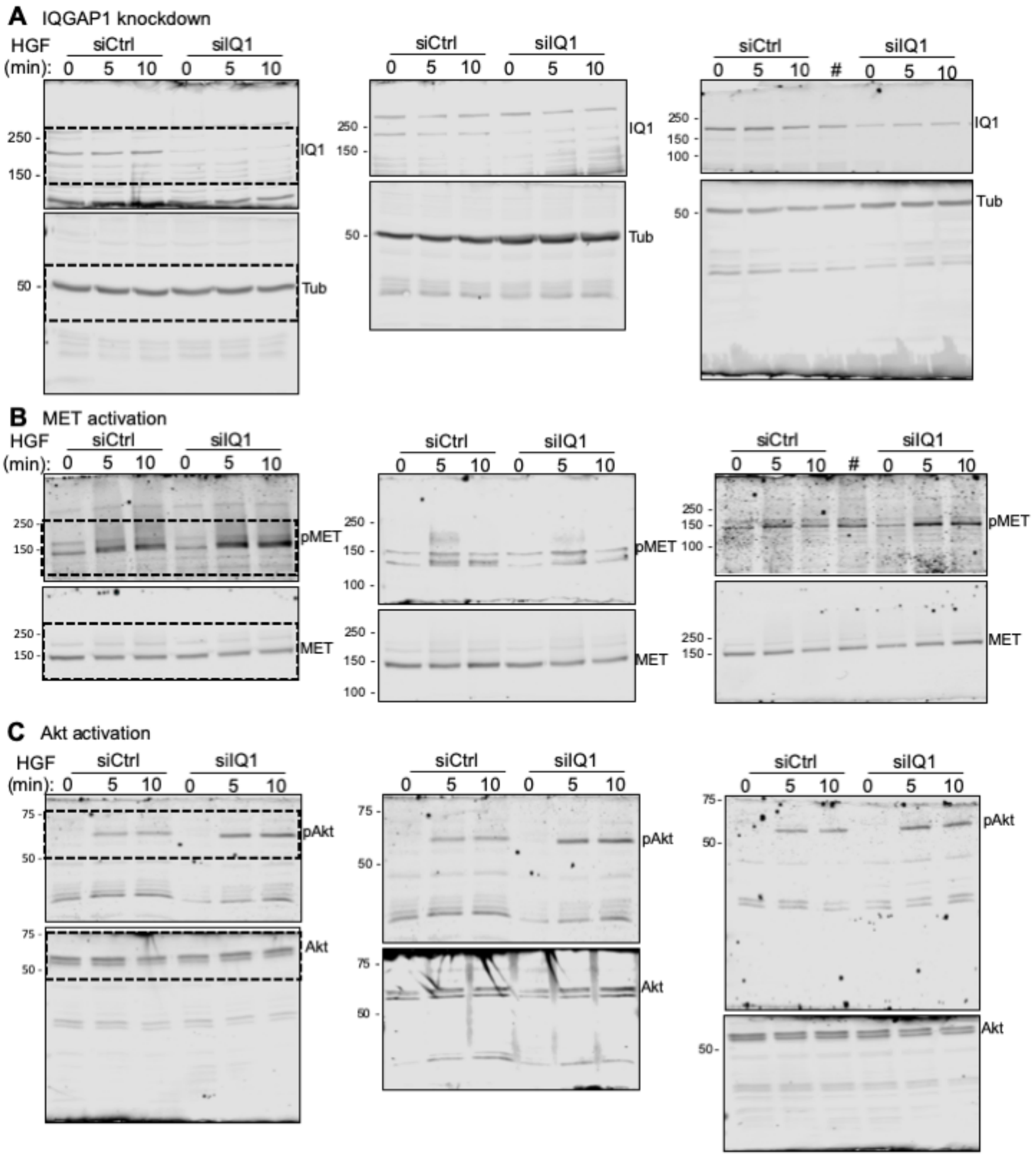
A IQGAP1 knockdown
HGF (min):
siCtrl
siIQ1
0 5 10 0 5 10
0 5 10 # 0 5 10
250
150
100
50
IQ1
Tub
B MET activation
pMET
MET
C Akt activation
75
50
pAkt
Akt

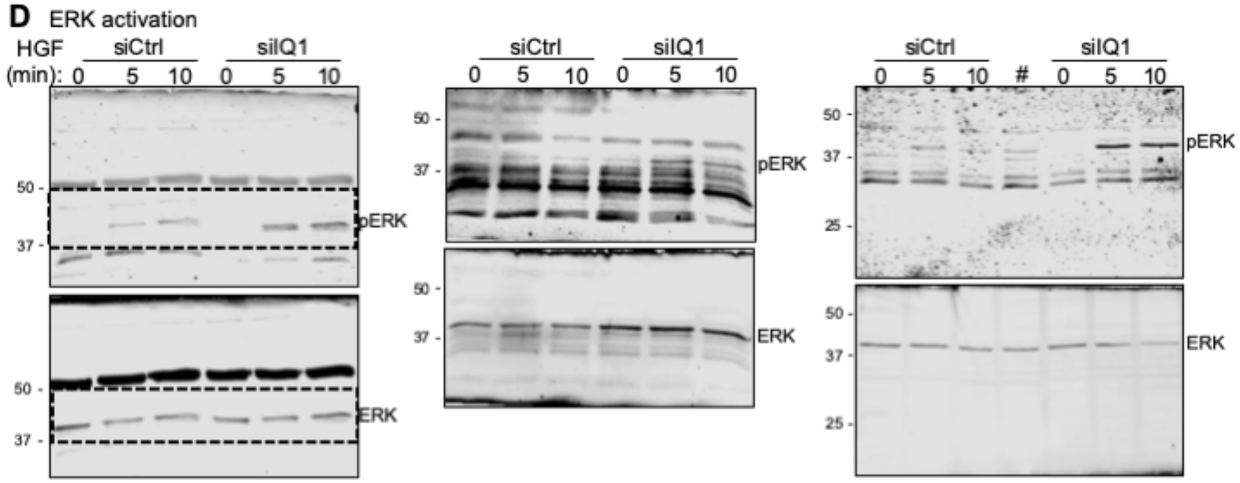

Figure S3. Raw immunoblots of Figure 2. The blots for each of the three independent biological replicates used in the quantitative analysis are shown. The dotted rectangles delineate the areas shown in Figure 2. # designates irrelevant lanes.

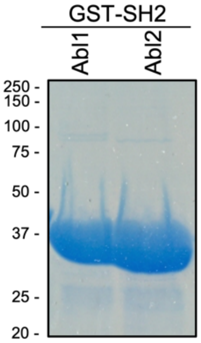

Figure S4. Purification of the GST-tagged SH2 domains of Abl1 and Abl2. The GST-tagged SH2 domains of Abl1 (39.55 kDa) and Abl2 (39.63 kDa) were expressed in *E. coli* and purified on glutathione-Sepharose beads. The SH2 constructs bound to the beads were analyzed by SDS-PAGE, followed by Coomassie blue staining.

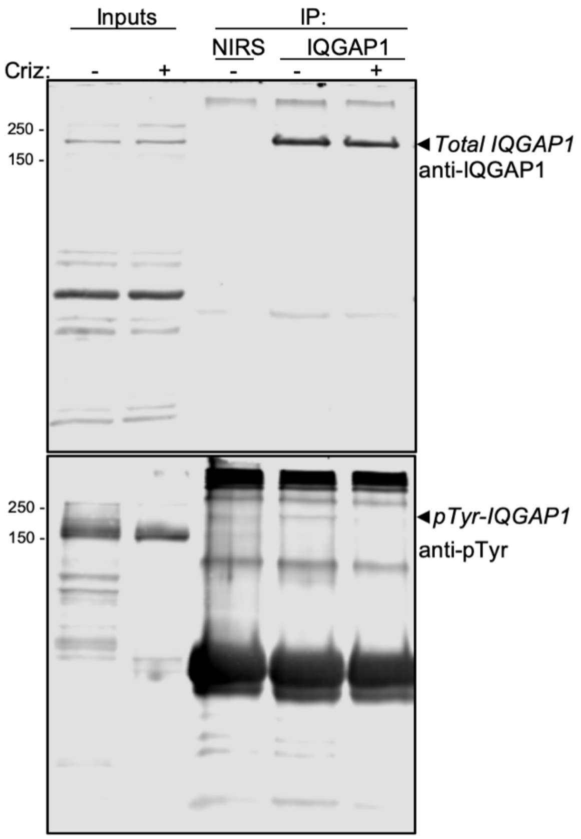

Figure S5. Crizotinib decreases MET-catalyzed tyrosine phosphorylation of IQGAP1 in H1993 cells. H1993 cells were treated with 100 nM crizotinib (criz, +) or vehicle DMSO (-) for 24 h. Cells were lysed and equal amounts of protein from cell lysates were subjected to immunoprecipitation (IP) with anti-IQGAP1 antibody. Control precipitation was carried out with non-immune rabbit serum (NIRS). Samples were resolved by SDS-PAGE and Western blotting, and probed with anti-IQGAP1 and anti-phosphotyrosine (pTyr) antibodies. Unfractionated cell lysates (Inputs) were processed in parallel. pTyr-IQGAP1 designates tyrosine-phosphorylated IQGAP1. Both images are from the same membrane.

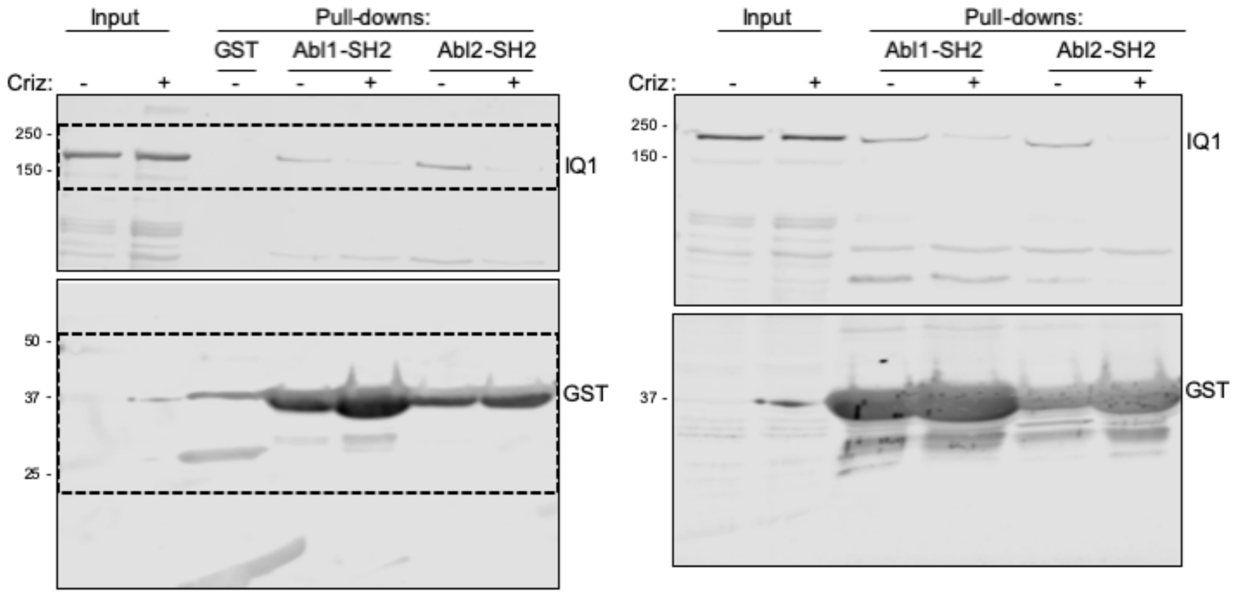

Figure S6. Raw immunoblots of Figure 4B. The blots for the two independent replicates used in the quantitative analysis are shown. The dotted rectangles delineate the areas shown in Figure 4B.

Abl1: Blots shown on Fig. 4D

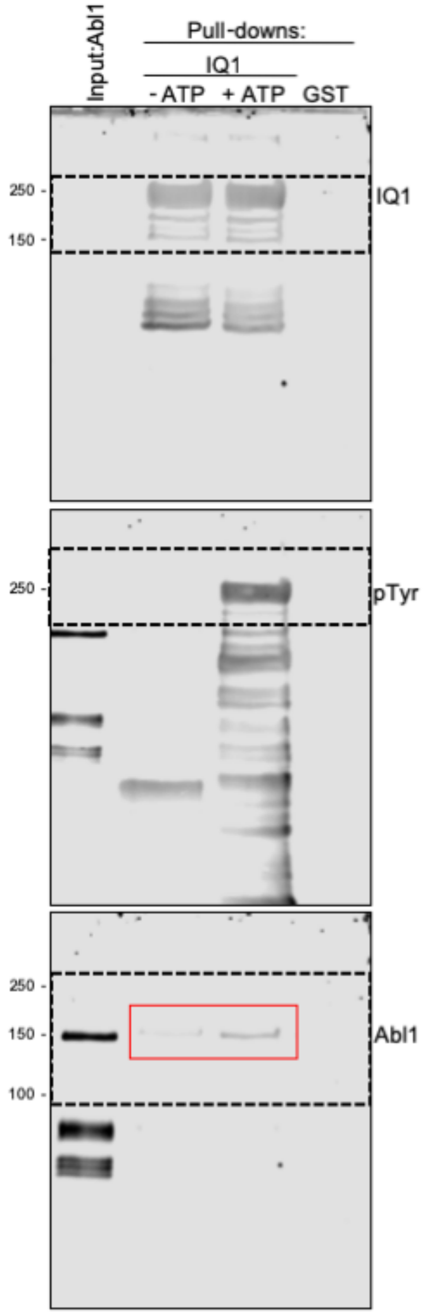

Abl1: Two other replicates used for quantitative analysis

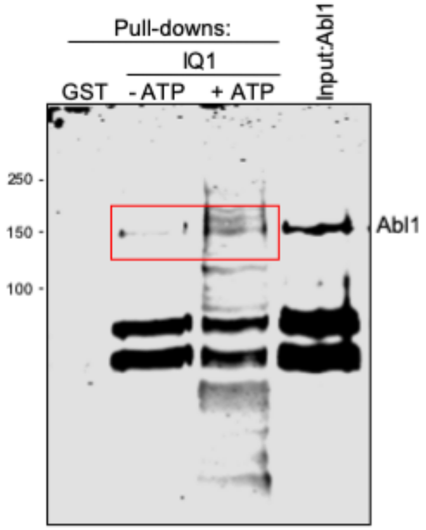

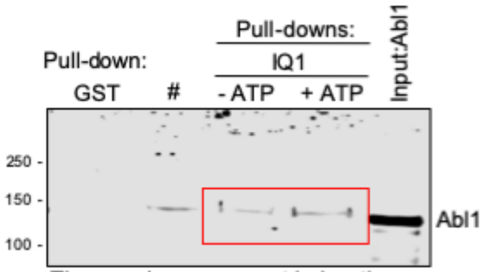

The membrane was cut below the 100 kDa marker.

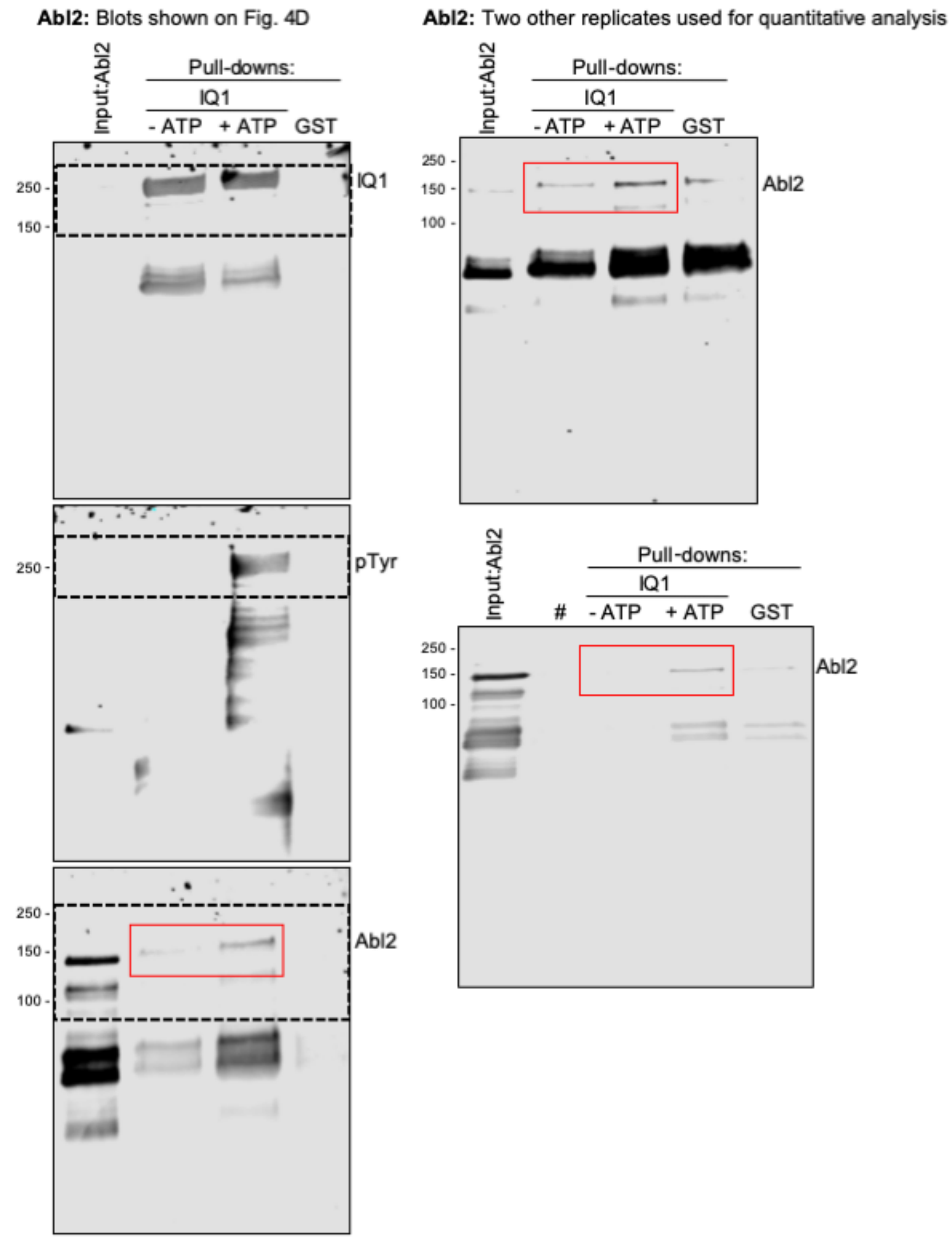

Figure S7. Raw immunoblots of Figure 4D. The blots of the three independent replicates used in the quantitative analysis are shown. The dotted rectangles delineate the areas shown in Figure 4B. The red rectangles delineate the bands that were quantified. # designates irrelevant lanes.

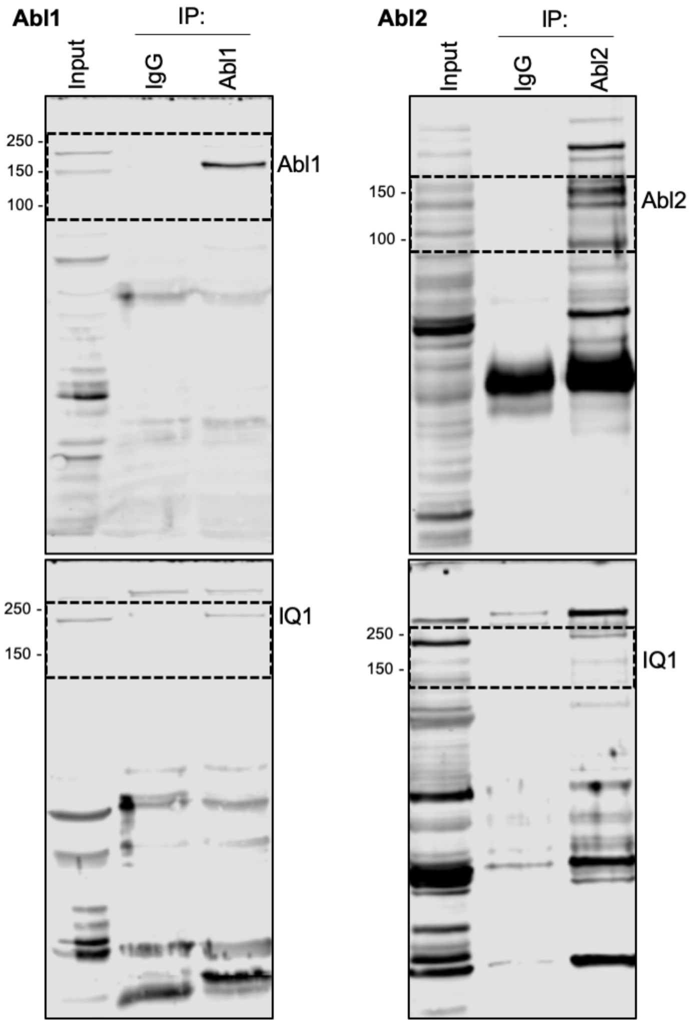

Figure S8. Raw immunoblots of Figure 5A. The dotted rectangles delineate the areas shown in Figure 5A.

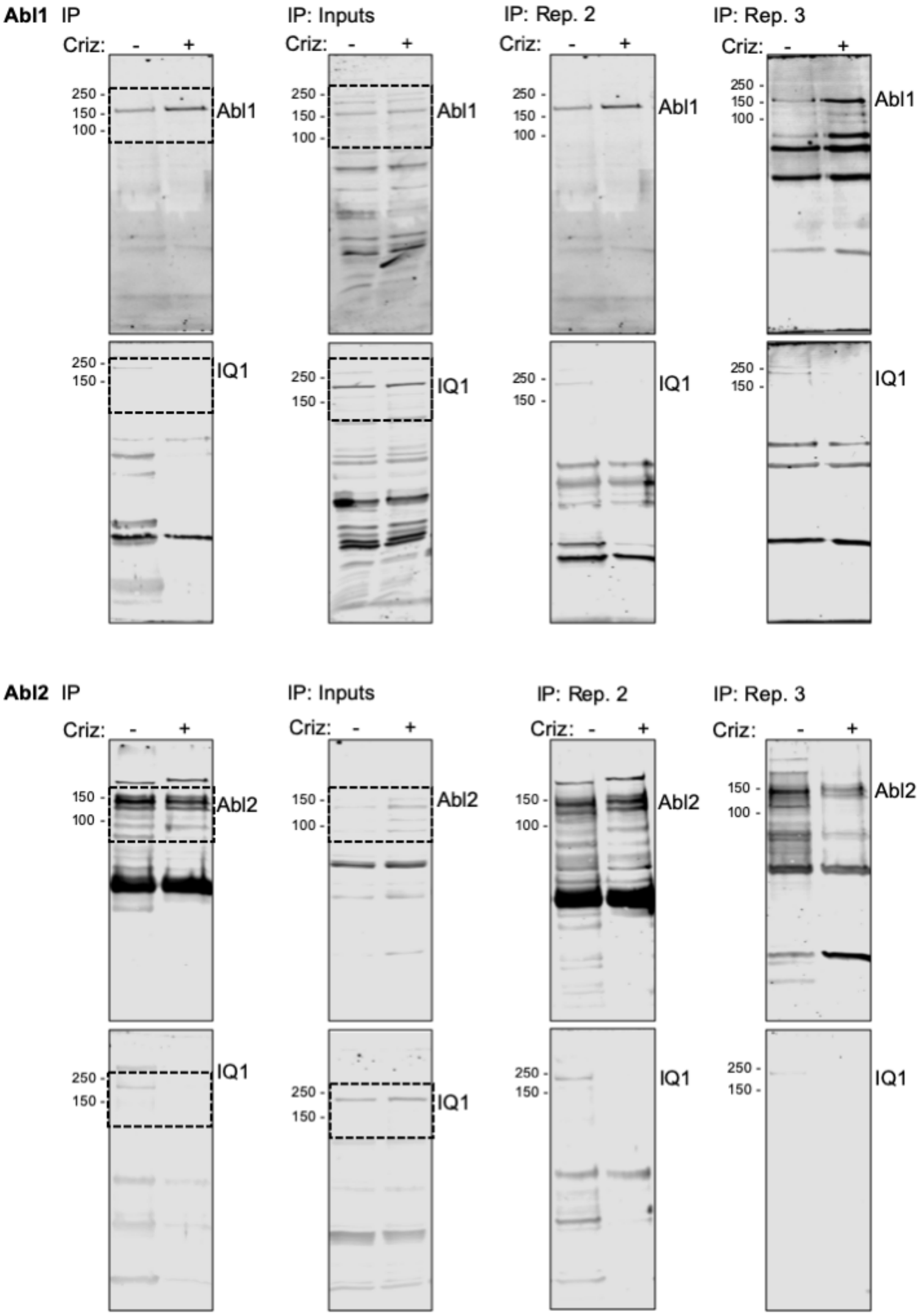

Figure S9. Raw immunoblots of Figure 5B. The blots of the three independent replicates used in the quantitative analysis are shown. The dotted rectangles delineate the areas shown in Figure 5B.

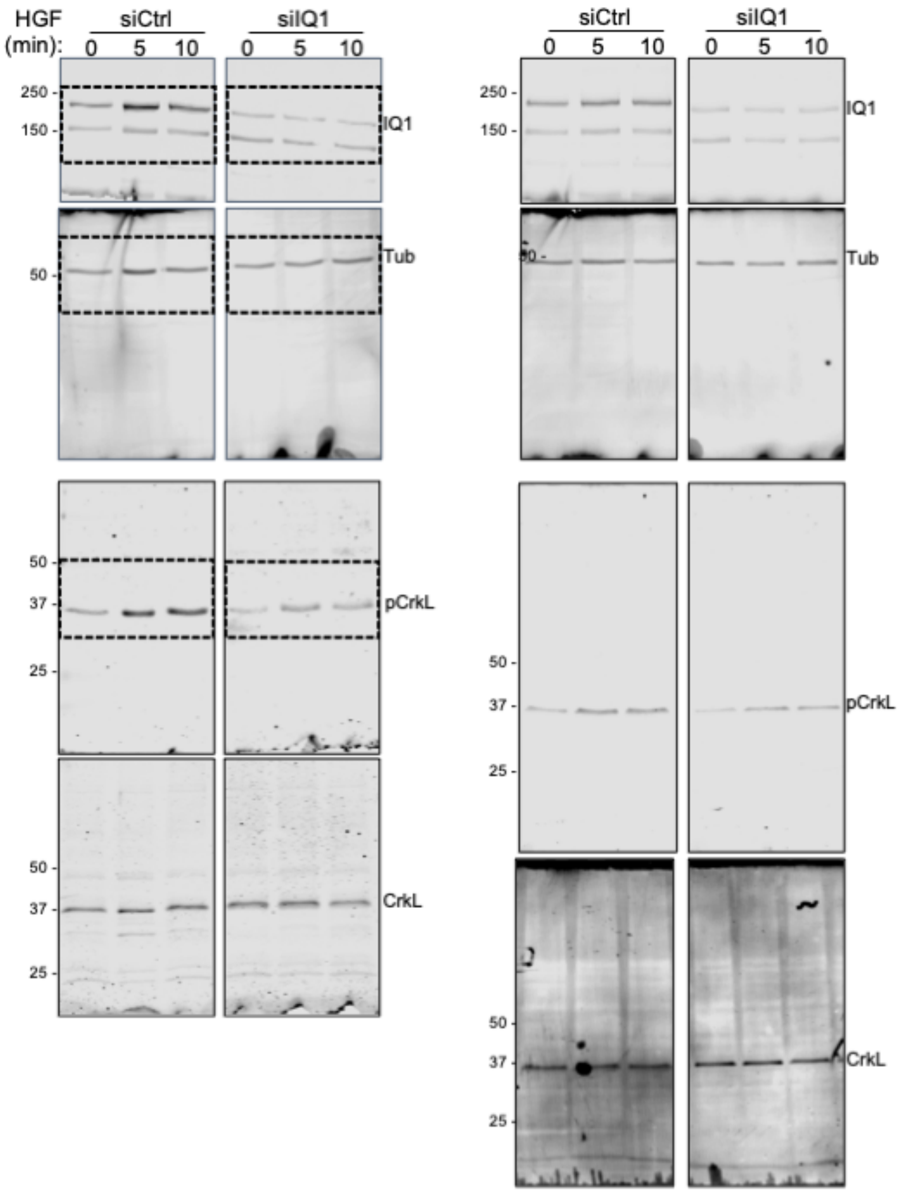
HGF (min):
siCtrl
siIQ1
0 5 10
250
150
IQ1
50
Tub
37
25
pCrkL
CrkL

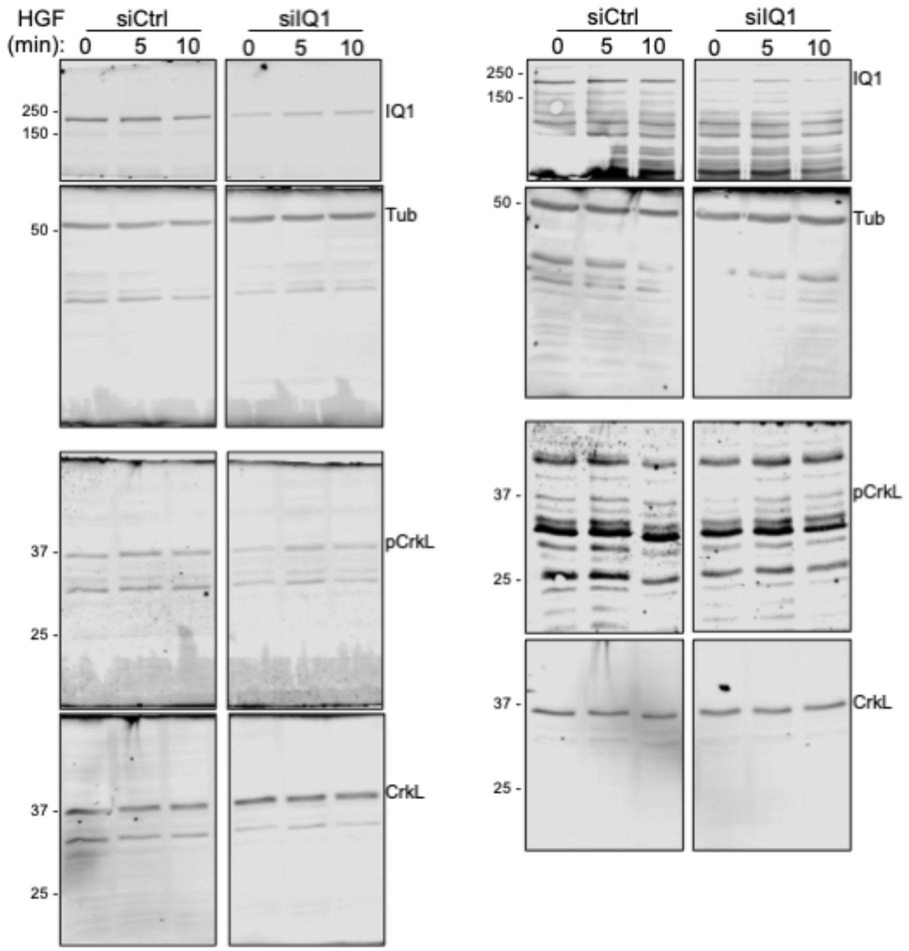

Figure S10. Raw immunoblots of Figure 6. The blots of each of the three independent biological replicates used in the quantitative analysis are shown. The dotted rectangles delineate the areas shown in Figure 6. All blots of each replicate are from the same cell lysates.
